# Juxta-articular tumoral calcinosis associated with the temporomandibular joint: a case report and concise review

**DOI:** 10.1186/s12903-019-0816-3

**Published:** 2019-07-09

**Authors:** Yang Sha, Kanglun Hong, Melvin Kang Ming Liew, Jing Li Lum, Raymond Chung Wen Wong

**Affiliations:** 1 Discipline of Oral and Maxillofacial Surgery, University Dental Cluster, National University Hospital Singapore, Singapore, Singapore; 2 Discipline of Oral and Maxillofacial Surgery, Faculty of Dentistry, National University of Singapore, Singapore, Singapore

**Keywords:** Tumoral calcinosis, Calcifying mass, Temporomandibular joint

## Abstract

**Background:**

Tumoral calcinosis is an uncommon clinicopathological condition which is characterized by the formation of calcium salt deposition in intra-articular or peri-articular soft tissues. It usually presents as a focal growth of hard tissue, either solitary or multiple, beneath the skin and connective tissue. Diagnostic techniques mainly include clinical and radiographic evaluation. The most commonly involved locations include the hip, elbow, shoulder and knee. Involvement of the head and neck regions are far less common. There have been 5 case reports of temporomandibular joint involvement in the literature so far.

**Case presentation:**

We present a case report which describes the diagnosis and management of a 59 year old female patient with chronic right temporomandibular joint pain and localized bony hard swelling over the right pre-auricular region. Patient retained normal range of motion and mouth opening. Computed tomography taken showed a radio-opaque juxta-articular ovoid mass over the right pre-auricular region in close proximity but not fused to the mandibular condyle. Surgical excision was performed for this swelling via a pre-auricular approach under general anaesthesia. Histological examination performed confirmed the diagnosis of tumoral calcinosis. Pain at the right temporomandibular joint was resolved after the surgery. Serum calcium and phosphate levels were normal in this patient.

**Conclusion:**

Surgical excision is the primary treatment modality for tumoral calcinosis. Tumoral calcinosis may be associated with elevated serum calcium and phosphate levels. In patients with elevated serum electrolytes, it is important to consider the overall systemic health in management of this condition. Management of serum electrolytes levels plays a role in reducing recurrence rates. This case report and review aims to discuss the diagnosis, treatment and overall systemic management of this rare condition.

## Background

In 1899, Duret first described a clinical case of calcifying cystic masses in the connective tissue adjacent to a joint space [1]. The term tumoral calcinosis (TC) was later proposed by Inclan and colleagues in 1943 for a disease characterized by large juxta-articular lobular calcified masses without visceral or skin calcifications in patients showing normal serum calcium and phosphate levels [2]. Since then, more than 200 cases of confirmed TC have been reported in the literature. It is a rare clinical condition commonly characterized by the deposition of calcium salt in intra-articular or peri-articular soft tissues. Clinically, TC presents as a focal growth of firm/hard lesion, either solitary or multiple, underneath skin and connective tissue and tends not to be attached to overlying soft tissue. Lesions progressively enlarge over a period of years, or less frequently, rapidly enlarge over a few months. Commonly asymptomatic at the initial stage, TC can attain a significant size and eventually lead to chronic pain, inflammation, limitation of function, with possible ulceration and fistulation of overlying skin. A yellowish or milky discharge is sometimes observed through the skin fistula. TC has been reported to affect populations in tropical and subtropical regions, especially in patients of African ethnicity. It has no significant sexual predilection and tends to occur in the first 2 decades of life [1–4, 7–10, 20]. It may be associated with elevated serum calcium and phosphate levels. Smack et al. classified TC into primary normophosphatemic, primary hyperphosphatemic or secondary sub-types based on pathogenesis [3].

The most commonly reported sites affected by TC are hip, elbow, shoulder and knee [3–5, 10]. Involvement of the cranium and head and neck region has rarely been reported. [11, 12, 21–24] G. Gal and colleagues conducted a review on head and neck manifestations of TC in 1994 [20]. They looked at 6 patients of Jewish-Yemenite origin with calcific masses of TC presenting around the facial region. Clinical and radiographic examination suggested correlation of TC with erythematous patches and maculopapular rash on the face and extremities, hoarseness of voice and angular cheilitis.

The first case of TC involving the temporomandibular joint (TMJ) was reported by K. Shirasuna in 1991 in a male Japanese patient [25]. Since then, only 4 more cases of diagnosed TC involving the TMJ region have been published in the literature to the best of the authors’ knowledge [26–29]. All 5 patients reported pain over the involved TMJ with varying degree of limited mouth opening. None of the patients exhibited familial traits or systemic risk factors known to cause TC. We report a case of calcifying mass located in close proximity to the right TMJ of a Chinese female patient complaining of chronic pain, which was diagnosed as TC.

## Case presentation

A 57-year-old Chinese female was referred to National University Hospital, Singapore in February 2016 for management of a hard swelling over her right TMJ. This patient suffered from chronic right TMJ pain for more than 5 years. She had consulted several physicians over the past few years, but could not obtain a definitive diagnosis. There was no prior treatment provided. Her past medical history includes surgical excision of left breast fibro-adenoma, recurrent upper urinary tract infection, stress urinary incontinence and infective colitis. She was on active follow up with the Otolaryngology department for hoarseness of voice. She was a non-smoker and non-drinker. There was no known history of any genetic disorder. The patient denied any history of trauma or infection to the jaw area. There were no other family members with a history of temporomandibular joint or jaw swelling.

On examination, a localized swelling was palpable over the right pre-auricular region which measured 15 mm by 20 mm. It was tender upon palpation and had a bony hard texture. The swelling appeared distinct from the TMJ capsule as it did not move when the right mandibular condyle translated anteriorly during mouth opening. On maximal mouth opening, the condylar translation at both TMJs was similar. The maximal mouth opening was normal. The overlying skin was normal in consistency and was not fixed to the swelling.

**Fig. 1:**
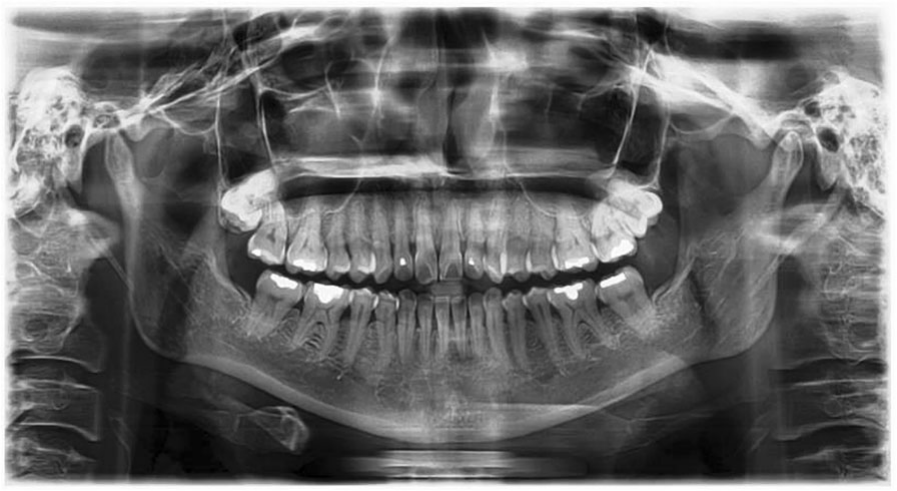
Dental panoramic tomogram of patient was taken on 18 Feb 2016. No significant abnormalities were detected

**Fig. 2:**
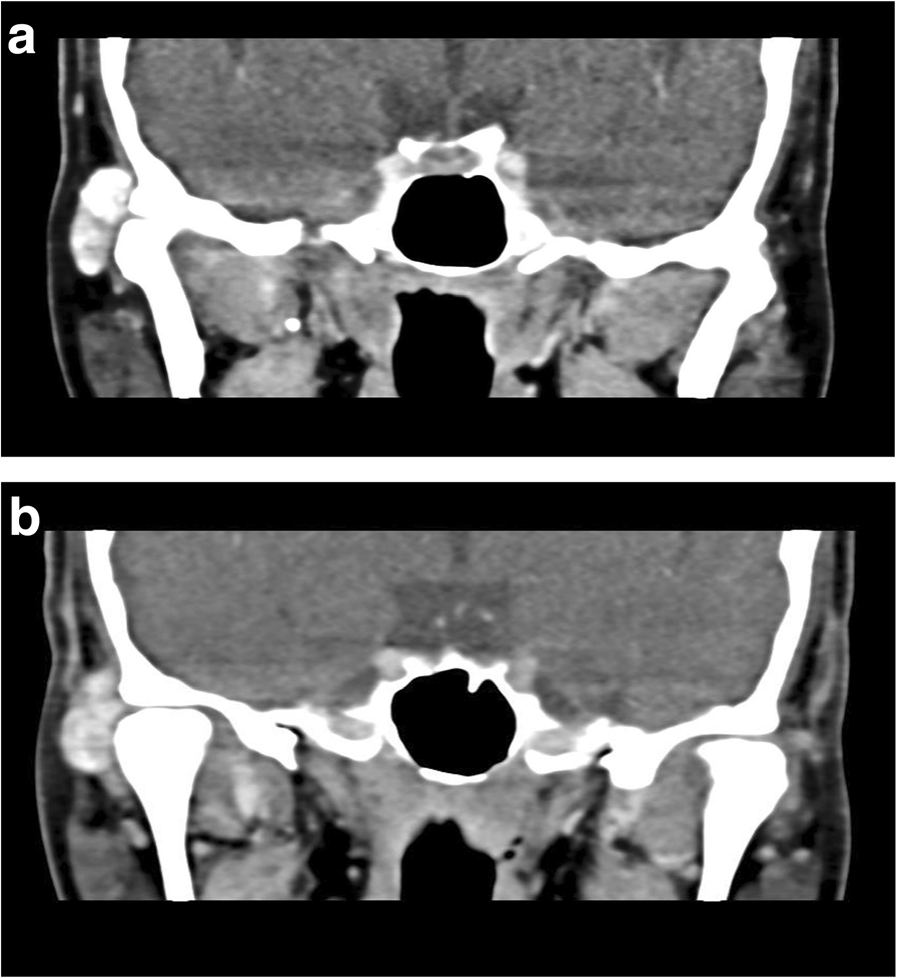
**a**. CT series coronal cut (soft tissue window), showing close association with lateral aspect of right condylar head. **b**. CT series coronal cut (soft tissue window), showing no bony erosion at right TMJ

A series of lab tests were ordered, including liver function test, renal function test and complete blood count. All results were being found to be within normal ranges. A dental panoramic tomogram (DPT) was taken (Fig. 1), but no distinct lesion could be observed at the right TMJ. Unenhanced computed tomography (CT) was taken subsequently (Fig. 2a-b). It revealed a sharply defined hyper-dense benign-looking ovoid mass measuring 10 mm by 13 mm by 20 mm within the subcutaneous layer over the right pre-auricular region. It was closely associated with the lateral aspect of the right TMJ. No bony erosion of the right TMJ was noted and both the joint capsule and mandibular condyle appeared normal.

The provisional diagnosis was tumoral calcinosis (TC) of the right TMJ. This was because the mass appeared distinct and was not contiguous with the right mandibular condyle. There was no erosion of the mandibular condyle. The joint space and capsule appeared preserved on CT. Other less likely differential diagnoses included synovial osteochondromatosis, synovial chondrosarcoma and osteochondroma.

**Fig. 3:**
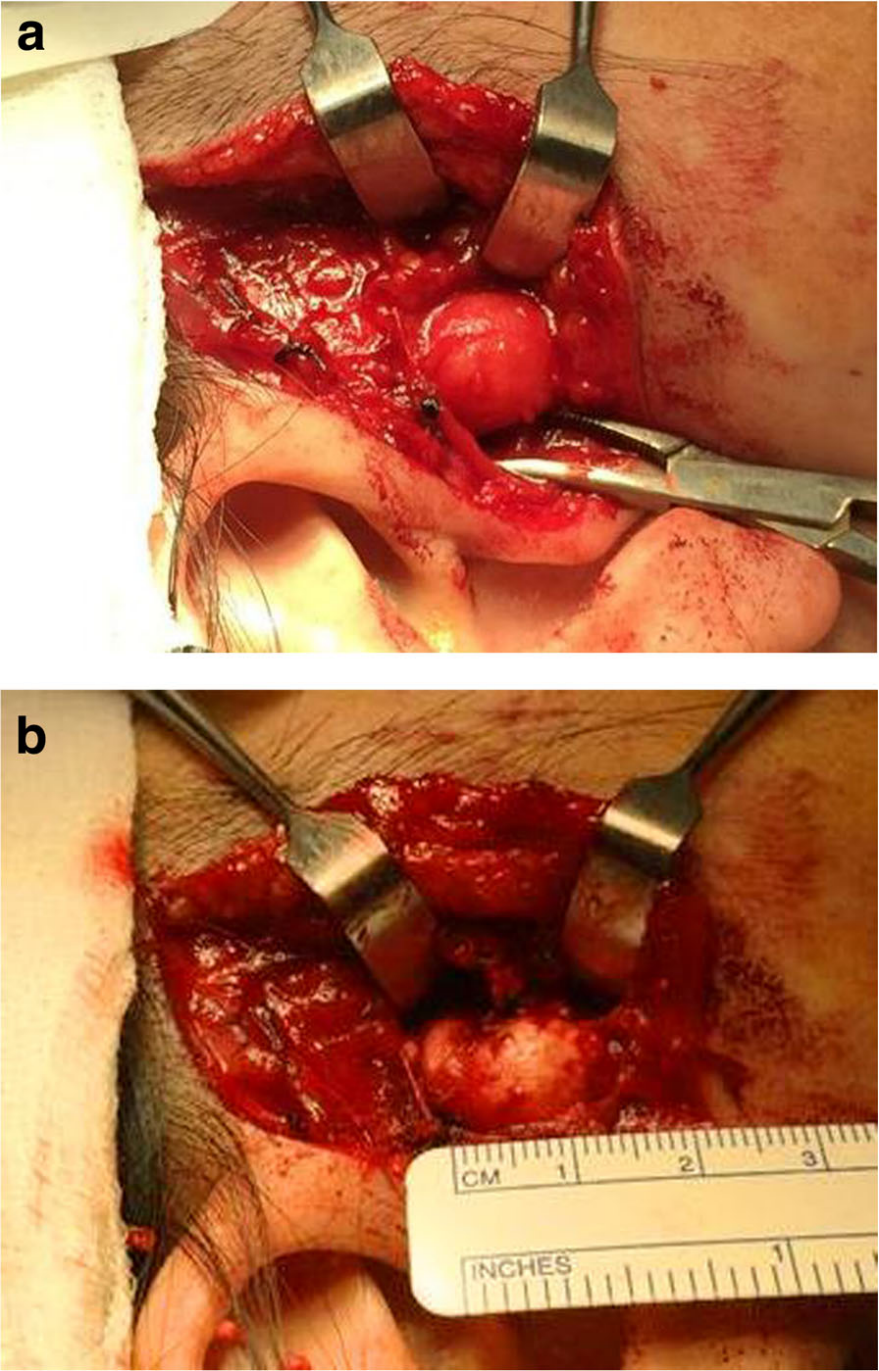
**a**. Incision of superficial layer of the deep temporal fascia with exposure of lesion. **b**. Dimensions of lesion

In view of the swelling and chronic pain of more than 5 years, she was advised to undergo surgical excision of the calcified mass. Subsequently, surgical excision of the calcified mass was performed through a pre-auricular approach under general anaesthesia in March 2016 (Fig. 3a-b). Intraoperatively, the mass which was excised was juxta-articular in terms of location. It was well-circumscribed, bony hard on palpation and had no fixation to the TMJ capsule.

**Fig. 4:**
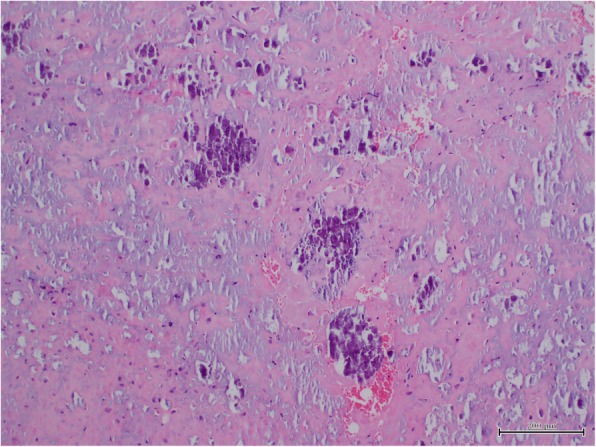
Histological section shows amorphous matrix that contains dense fibrous tissue and deposits of calcified material. Some inflammatory elements are present. (Hematoxylin-Eosin stain, magnification × 10)

Histological examination of the specimen reported nodular fragments of amorphous pink matrix and fibrous tissue with calcified areas. There were areas of foreign body reaction. Figure 4 there was no evidence of epithelial proliferation, atypia, neoplasm or malignancy. The specimen tested negative to Congo-red stain. Refractile crystals were not identified. The histological findings were compatible with tumoral calcinosis.

The post-operative recovery was uneventful. Pain at the right pre-auricular region was resolved after surgical excision of the swelling. There was no limitation of function of the right TMJ. Maximal mouth opening was normal and the patient could eat well. Upon histological confirmation of TC, a baseline serum electrolyte test was ordered for this patient. The baseline serum calcium and phosphate levels were normal.

The patient was reviewed regularly for 3 months. However, she subsequently defaulted on her follow-up appointments after the review in April 2016. She eventually returned for a review on September 2017, 17 months after surgical excision. There was no clinical recurrence noted. The pre-auricular skin wound healed with minimal scarring. She reported minor sensory deficit in the right pre-auricular region with a Visual Analog Scale score of 1/10. Other than the minor sensory deficit, there were no other adverse outcomes. She was able to functional well without any limitation or pain. Serum electrolytes were tested again and levels were normal. Further follow-up appointments were arranged. However, the patient defaulted on the subsequent appointments.

### Case report timeline

**Table:** 

Relevant Past Medical History and Interventions	
➢ 57 years old Female Chinese ➢ Past medical history included surgical excision of left breast fibro-adenoma, recurrent upper urinary tract infection, stress urinary incontinence and infective colitis. Patient was also on follow up with the Otolaryngology department for hoarseness of voice. ➢ Presented with chronic right TMJ pain for more than 5 years, as well as localized swelling over right pre-auricular region. Previously consulted multiple physicians but no definitive diagnosis was obtained. No prior treatment was provided.

**Table:** 

Date	Summary from Initial and Subsequent Visits	Diagnostic Assessment	Therapeutic Interventions
February 2016 to March 2016	➢ First seen in *February 2016*. ➢ Chief complaint was that of chronic right TMJ pain and hard swelling over right pre-auricular region. ➢ On examination, the swelling measured 15x20mm. It did not move with the mandibular condyle on maximal mouth opening. ➢ Provisional diagnosis was that of Tumoral Calcinosis associated with the right TMJ.	➢ Blood tests including liver function test, renal function test and complete blood count were normal. ➢ Dental pantomogram was taken, but no distinct lesion was observed. ➢ Computed Tomography taken revealed a well-defined hyper-dense mass at the right pre-auricular region.	➢ Surgical excision of the calcified mass was performed in *March 2016*. ➢ Histological examination confirmed diagnosis of tumoral calcinosis (TC).
April 2016 to July 2016	Post-operative reviews: ➢ Pain at the right pre-auricular region was resolved. ➢ TMJ function was normal.	➢ The baseline serum calcium and phosphate levels were normal.	➢ Nil
September 2017	Final review: ➢ No clinical recurrence was noted. ➢ TMJ function was normal ➢ Patient subsequently defaulted on further follow-up appointments.	➢ Serum calcium and serum phosphate were tested and were normal. ➢ Patient belonged to the Normophosphatemic TC sub-type (Smack et al [3]).	➢ Nil

## Discussion

Tumoral calcinosis is a rare disease entity and its involvement at the TMJ region remains scarce. Common differential diagnosis for a hard tissue growth around the pre-auricular region includes synovial osteochondromatosis, synovial chondrosarcoma, osteochondroma, and osteochondrosarcoma. Synovial chondromatosis is a benign condition characterized by synovial membrane proliferation and metaplasia. It presents as calcifying nodules of cartilage within the synovial membrane of the TMJ and usually affects a unilateral joint space. It is often associated with trauma. The loose calcified nodules may increase in numbers with time and can eventually occupy the entire joint space. Like TC, synovial chondromatosis can result in pain, swelling and limitation of range of motion of the TMJ. Synovial chondrosarcoma is a very rare malignancy that can arise from synovial chondromatosis or de novo within the synovial membrane. Synovial chondrosarcoma involvement of the TMJ is rare.

Osteochondroma is more commonly associated with the axial skeleton and less commonly associated with the facial bones. It is a slow-growing benign tumour which causes enlargement of the mandibular condyle with time. It results in limitation of mouth opening, malocclusion and TMJ dysfunction. Osteochondroma, despite a similar presentation on plain radiographs, exhibit a characteristic cartilage-capping over the bony lesion which appears to be contiguous with periosteum of the affected bone.

On the contrary, TC is not contiguous with the periosteum of the affected bone. It may present as intra-articular or juxta-articular lesions. Of the 5 reported cases of TMJ involvement, 3 of them were intra-articular [27–29] and 2 of them were juxta-articular [25, 26]. Intra-articular TC commonly leads to limitation of mouth opening [28, 29]. Unlike intra-articular TC, juxta-articular TC less commonly leads to limitation of mouth opening as joint movement is unaffected. In this patient, the lesion was juxta-articular as it was not contiguous with the mandibular condyle and TMJ capsule. Mouth opening was normal in this patient.

The diagnosis of TC is primarily based on imaging. Plain radiographs depict amorphous, multi-lobulated radio-opaque masses adjacent to the mandibular condyle. In this patient, the DPT did not demonstrate the presence of the lesion. This could be attributed to the fact that the lesion was not as radio-opaque when compared to its surrounding bone, owing to its lower density. In addition, its presence on the DPT may be obscured by the skull base due to its location. This case report highlights the difficulty in diagnosing the lesion using the DPT. In patients where the swelling is not highly noticeable during clinical examination, it is possible that such a lesion can be missed or undiagnosed.

This highlights the need for CT for proper evaluation of such lesions. CT scan is critical for diagnosis as it provides the precise location, size and relations to the surrounding tissues. Of the 5 reported cases of TMJ involvement, 4 were diagnosed using CT scans [25, 26, 28, 29]. CT images may show cystic loculi with fluid-fluid levels caused by calcium layering. It may also appear homogenous when there is decreased activity in the quiescent stage. Erosion or osseous destruction is consistently absent. This was consistent with the CT findings for this patient where there was no erosion of the mandibular condyle or surrounding bony structures.

Of the 5 reported cases of TMJ involvement, only 1 was diagnosed using magnetic resonance imaging [27]. Magnetic resonance imaging is less commonly used as compared to CT. It usually shows non-homogeneous high signal intensity on T2-weighted sequences and non-homogeneous low signal intensity on T1-weighted sequences. Scintigraphy scanning using radiolabeled phosphate compounds (technetium-99 methylene diphosphate) is useful for diagnosing multiple lesions, newly formed lesions and monitoring of lesion activitity [10].

Histological features of TC are distinctively different from the rest. Idiopathic calcifications are evident with positive calcium stains using von Kossa’s method, without involvement of cartilage transformation [3, 4]. Calcifications in TC are also initiated in histiocytic aggregates and are not directly within soft tissue or cartilage. In addition, there are characteristic compartments that contain both calcifications and liquid milky/chalky contents [31]. The lesion is often encased by a dense fibrous capsule, frequently separated by fibrous trabeculae, with scattering of inflammatory cells [3–6].

The exact pathogenesis of tumoral calcinosis remains unconfirmed till today. Several theories have been proposed in the literature, alluding to its possible association with systemic hyperphosphatemia in patients receiving chronic haemodialysis and patients with potential underlying familial traits that lead to mutation of genes involved in phosphate metabolism, e.g.FGF-23 (Fibroblast Growth Factor - 23) and GALNT-3 (Polypeptide N-Acetylgalactosaminyltransferase - 3), with presence of associated mechanical injury [3, 4, 6, 10, 13–17].

A commonly recognized classification for TC was introduced by Smack et al., who proposed a classification based on possible pathogenesis after retrospectively reviewing 122 cases of TC [3] (Table 1).

**Table 1:** Adapted from Smack et al. [3]

Classification	Main demographic	Familial patterns	Common Sites	Serum Electrolyte Level	Clinical Presentation
Primary Normophosphatemic TC	- Onset before 2nd decade (63%) - Mostly involve patients in tropical or subtropical regions - No sexual predilection	- Nil familial pattern	- Hip (31%) - Elbow (24%) - Knee (16%)	- Normal serum phosphate - Normal serum calcium	- Solitary calcification predominates (66%) - History of trauma - Milky fluid of calcification on incision are common
Primary Hyperphosphatemic TC	- Onset before 2nd decade (82%) - No sexual predilection - Higher frequency in blacks and men	- Strong familial pattern - Most patients are siblings (74%)	- Hip (37%) - Elbow (27%) - Shoulder (23%)	- Elevated serum phosphate - Elevated serum calcium	- Multiple calcifications predominate (74%) - Milky fluid of calcification on incision in all cases reviewed
Secondary TC	- Onset before 2nd decade (51%) - Higher frequency in whites and women - Chronic renal failure is the most common identifiable condition	- Some familial pattern	- Hip (29%) - Elbow (24%) - Shoulder (17%)	- Elevated serum calcium	- Multiple calcifications predominate (80%) - Milky fluid of calcification on incision in all cases reviewed

Veress in 1976 classified TC into active and inactive phases based on differential histological appearances of the excised lesion [7]. It was reported that in the active phase of TC, the cystic lesion was filled with fluids and basophilic material, with occasional appearance of degenerated collagen. The surrounding granulation tissue was populated with both mononuclear cells and giant cells. Endothelial cell proliferation and capillaries were often noted. In the inactive phase, TC cysts were filled with calcium deposits, with or without degenerated collagen. Surrounding granulation tissue was largely replaced by acellular scar tissue with absence of lipids.

Slavin et al. reported that the histology is identical across all three sub-groups of TC as defined by Smack et al. [31]. Slavin et al. more recently described the histological development of the lesion based on three stages. In the first stage, nodular and linear clusters of foamy histiocytes aggregate around small blood vessels. These histiocytes, located in tendon or fascia, extend into adjacent tendon, fascia, muscle, and skin. The histiocytic aggregates undergo granular necrosis. Together with degenerative alterations in collagen, cavities or locules are created. The second stage is the calcifying phase. The disintegrating histiocytes in the newly formed locules exhibit membrane-bound punctuate cytoplasmic calcifications, whereas admixed giant cells exhibit larger plate-like calcifications. A calcifying front forms at the border of the surviving locular lining cells and the locular calcifications. It consists of Von Kossa positive sand-like and plate-like calcifications. These calcifications coalesce centrally to form larger calculi. The third stage is characterized by disruption and fibrosis of the locular septa, disappearance of locular lining cells, and coalescence. The lesions become quiescent when there is decrease in calcification and collagenolysis. The lesions end with fibrosis which surrounds the TC lesions [30, 31].

Slavin et al. postulated that the development of TC requires 2 conditions – presence of injury i.e. a juxta-articular or peri-osseous injury and a metabolic abnormality i.e. elevated calcium or phosphate levels. TC appears to be triggered by presence of bleeding caused by minimal repetitive trauma, resulting in an aggregation of foamy histiocytes. These are in turn transformed into cystic cavities due to degeneration of collagen and granular necrosis of the cells. Following this, a calcifying process in relation to elevated serum phosphorus facilitates the nucleation of hydroxyapatite into cells and extracellular matrix materials. However, collagenolysis due to proteolytic enzymes produced from disintegrating histiocytes prevents functional bone formation [30, 31]. In patients with non-familial TC, hyperphosphatemia may be caused by locally induced causes, such as tissue trauma releasing phosphate from injured muscle cells into the extracellular space.

In the 5 reported cases of TMJ involvement, all patients showed normal serum calcium and phosphate levels. Upon histological confirmation of TC in this patient, a baseline serum electrolyte test was performed. The baseline serum electrolyte tests including calcium and phosphate levels were normal. Follow-up serum electrolyte levels which were tested 17 months post-surgery were being found to be within normal limits as well. Based on the medical history, clinical presentation and normal serum electrolytes, it was concluded that this patient belonged to the Primary Normophosphatemic TC sub-type. This patient presented with a solitary TC, which was also consistent with the presentation of solitary calcifications that predominate in the Primary Normophosphatemic TC sub-type. In addition, no familial patterns were evident in this patient, consistent with this particular sub-type.

For primary variety TC sub-types (Primary Normophosphatemic and Primary Hyperphosphatemic), the primary treatment modality is surgical excision [32]. This was the treatment modality successfully employed for this patient. During surgery, TC lesions may show a cystic nature with white and yellow chalky material formed by calcium hydroxyapatite crystals, calcium carbonate and calcium phosphate. The excised sample was not cut open to reveal the interior contents intraoperatively, prior to sending for histological examination. This was because the surgeons wanted to preserve an intact capsule for histological examination. Hence, the gross appearance of the interior contents could not be verified intra-operatively.

For secondary TC sub-type patients with chronic renal failure and haemodialysis, surgery may not be the first option due to the presence of multiple co-morbidities and higher risk of recurrence. In such patients, medical treatment may have a role. Treatment modalities such as calcium-depleted electrolytic bath [13], phosphate binders [14, 16], calcitonin, bisphosphonate [16] and diet restriction of phosphate were used to restore phosphate and calcium metabolism. This has been shown to reduce size of TC lesions to various degrees. Topical, intravenous and intra-lesional injections of sodium thiosulfate were also reported with limited success in some studies. The mechanism of action still remains unknown [18, 19].

According to Smack et al., the risk of recurrence after surgical excision for patients in the Primary Normophosphatemic TC sub-type is low [3]. However, the risk of recurrence for patients in the Primary Hyperphosphatemic TC and Secondary TC sub-types are higher [1–3, 6, 10, 16, 17, 26–29]. In the Primary Normophosphatemic TC sub-type, the lower recurrence rate could be due to normal serum calcium and phosphate levels. The higher recurrence rate in the Primary Hyperphosphatemic TC sub-type and Secondary TC sub-type could be due to the systemic influence caused by elevated serum calcium and phosphate levels.

## Conclusion

Tumoral calcinosis associated with the temporomandibular joint is a rare condition. Proper diagnosis requires detailed history taking, clinical examination, radiographic investigations and baseline serum electrolytes. Computed tomography is preferred over Dental Panoramic Tomogram as such calcifying lesions may not be clearly seen in DPT images as exemplified by this case. Computed tomography provides a detailed assessment of the location, size and relations to surrounding tissues. Surgical excision is usually the treatment of choice for Primary Normophosphatemic TC and Primary Hyperphosphatemic TC sub-types. Medical therapy such as phosphate binders, calcitonin can play a role in management of Secondary TC sub-type. It is important to consider the overall systemic health in patients belonging to Primary Hyperphosphatemic TC and Secondary TC sub-types, due to raised serum electrolytes. Correction of the serum electrolytes plays a crucial role in reducing the risk of recurrence of these lesions. It is advisable to refer patients belonging to the Primary Hyperphosphatemic TC or Secondary TC sub-types to the appropriate physician for evaluation of underlying causes in relation to raised serum electrolytes. Monitoring for recurrence of lesions is important for the Primary Hyperphosphatemic TC or Secondary TC sub-types, due to the higher recurrence rates. However, there is no commonly accepted protocol for long term follow-up of TC due to the rarity of this condition. We suggest a yearly follow-up for all three sub-types of TC. In particular, there should be longer follow-up periods for the Primary Hyperphosphatemic TC and Secondary TC sub-types.

## Data Availability

Not applicable

## Abbreviations

DPT: Dental panoramic tomogram
TC: Tumoral calcinosis
TMJ: Temporomandibular joint

## References

[1] Duret MH (1899). Tumerus multiples et singerlaires des courser sereuses. Bull Mem Sot Anat.

[2] Inclan A, Leon PP, Camejo M (1943). Tumoral calcinosis. J Am Med Ass.

[3] Smack D, Norton SA, Fitzpatrick JE (1996). Proposal for a pathogenesis-based classification of tumoral calcinosis. Int J Dermatol.

[4] Knowles SA, Declerck G, Anthony PP (1983). Tumoral calcinosis. Br J Surg.

[5] McClatchie S, Bremner AD (1969). Tumoral calcinosis – an unrecognized disease. Br Med J.

[6] Braun W, Mayr E, Kundel K, Rüter A (1996). Tumorous calcinosis: a disease of its own?. Arch Orthop Trauma Surg.

[7] Veress B, Malik MO, El Hassan AM (1976). Tumoural lipocalcinosis: a clinicopathological study of 20 cases. J Pathol.

[8] Palmer PE (1966). Tumoral calcinosis. Br J Radiol.

[9] Thomson JG (1966). Calcifying collagenolysis (tumoral calcinosis). Br J Radiol.

[10] Fathi I, Sakr M (2014). Review of tumoral calcinosis: a rare clinico-pathological entity. World J Clin Cases.

[11] Jakka S, Narayan R, Mishra M, Rana F, Laik JK (2017). Idiopathic Tumoral calcinosis - rare Clinico pathological entity: a report of two cases. J Clin Diagn Res.

[12] Di Serafino M, Gioioso M, Severino R, Lisanti F, Rocca R, Sorbo P, Maroscia D (2017). The idiopathic localized tumoral calcinosis: the “chicken wire” radiographic pattern. Radiol Case Rep.

[13] Ibrahim Montasser D, Issouani J, Hassani M, Kabbaj D (2017). Tumoral calcinosis: diffuse multifocal form in hemodialysis patients. Two case reports. Orthop Traumatol Surg Res.

[14] Nishime K, Takahashi H (2016). Acute tumoral calcinosis due to severe hyperphosphatemia in a maintenance hemodialysis patient. CEN Case Rep.

[15] Moe OW (2014). Familial tumoral calcinosis: a valuable vehicle for discovery. Nephrol Dial Transplant.

[16] Folsom LJ, Imel EA (2015). Hyperphosphatemic familial tumoral calcinosis: genetic models of deficient FGF23 action. Curr Osteoporos Rep.

[17] Lykoudis EG, Seretis K, Ristanis S (2012). Huge recurrent tumoral calcinosis needing extensive excision and reconstruction: report of a rare case and brief literature review. Aesthet Plast Surg.

[18] Goossens J, Courbebaisse M, Caudron E, Bahans C, Vacquerie V, Melchior J, Salle PV, Moesch C, Daudon M, Frocht V, Richette P, Ea HK, Guigonis V (2017). Efficacy of intralesional sodium thiosulfate injections for disabling tumoral calcinosis: two cases. Semin Arthritis Rheum.

[19] Mageau A, Guigonis V, Ratzimbasafy V, Bardin T, Richette P, Urena P, Ea HK (2017). Intravenous sodium thiosulfate for treating tumoral calcinosis associated with systemic disorders: Report of four cases. Joint Bone Spine.

[20] Gal G, Metzker A, Garlick J, Gold Y, Calderon S (1994). Head and neck manifestations of tumoral calcinosis. Oral Surg Oral Med Oral Pathol..

[21] Hunter IP, MacDonald DG, Ferguson MM (1973). Developmental abnormalities of the dentine and pulp associated with tumoral calcinosis. Br Dent J.

[22] Burkes EJ, Lyles KW, Dolan EA, Giammara B, Hanker J (1991). Dental lesions in tumoral calcinosis. J Oral Pathol Med.

[23] Witcher SL, Drinkard DW, Shapiro RD, Schow CE (1989). Tumoral calcinosis with unusual dental radiographic findings. Oral Surg Oral Med Oral Pathol.

[24] Marinho RO, Anderson GP, Warren AY (1999). Tumoral calcinosis in the premaxillary region. Oral Surg Oral Med Oral Pathol Oral Radiol Endod.

[25] Shirasuna K, Sugiyama M, Yasui Y (1991). Tumoral calcinosis around the mandibular condyle. Int J Oral Maxillofac Surg.

[26] Zanetti U, Derada Troletti G, Burlini D, Rossi D (1994). Tumoral calcinosis: a case report. Int J Oral Maxillofac Surg.

[27] Sledz K, Ortiz O, Wax M, Bouquot J (1995). Tumoral calcinosis of the temporomandibular joint: CT and MR findings. AJNR Am J Neuroradiol.

[28] Yang C, Qiu WL (2002). Bilateral discal tumoral calcinosis of the temporomandibular joint. J Oral Maxillofac Surg.

[29] Dimitroulis G (2004). Tumoral calcinosis of the articular disc of the temporomandibular joint: a rare entity. J Oral Maxillofac Surg.

[30] Slavin RE, Wen J, Kumar D, Evans EB (1993). Familial tumoral calcinosis. A clinical, histopathologic, and ultrastructural study with an analysis of its calcifying process and pathogenesis. Am J Surg Pathol.

[31] Slavin RE, Wen J, Barmada A (2012). Tumoral calcinosis--a pathogenetic overview: a histological and ultrastructural study with a report of two new cases, one in infancy. Int J Surg Pathol.

[32] Steinbach LS, Johnston JO, Tepper EF, Honda GD, Martel W (1995). Tumoral calcinosis: radiologic-pathologic correlation. Skelet Radiol.

